# Liver Regeneration by Hematopoietic Stem Cells: Have We Reached the End of the Road?

**DOI:** 10.3390/cells11152312

**Published:** 2022-07-27

**Authors:** Elena Konstantina Siapati, Maria G. Roubelakis, George Vassilopoulos

**Affiliations:** 1 Faculty of Health and Rehabilitation Sciences, Metropolitan College, 15125 Athens, Greece; 2 Laboratory of Biology, Medical School, National and Kapodistrian University of Athens, 11527 Athens, Greece; roubel@med.uoa.gr; 3 Centre of Basic Research, Biomedical Research Foundation of the Academy of Athens (BRFAA), 11527 Athens, Greece; 4 Department of Hematology, UHL, University of Thessaly Medical School, 41110 Larissa, Greece

**Keywords:** HSC, liver disease, stem cell therapy, transdifferentiation, fusion, clinical trial

## Abstract

The liver is the organ with the highest regenerative capacity in the human body. However, various insults, including viral infections, alcohol or drug abuse, and metabolic overload, may cause chronic inflammation and fibrosis, leading to irreversible liver dysfunction. Despite advances in surgery and pharmacological treatments, liver diseases remain a leading cause of death worldwide. To address the shortage of donor liver organs for orthotopic liver transplantation, cell therapy in liver disease has emerged as a promising regenerative treatment. Sources include primary hepatocytes or functional hepatocytes generated from the reprogramming of induced pluripotent stem cells (iPSC). Different types of stem cells have also been employed for transplantation to trigger regeneration, including hematopoietic stem cells (HSCs), mesenchymal stromal cells (MSCs), endothelial progenitor cells (EPCs) as well as adult and fetal liver progenitor cells. HSCs, usually defined by the expression of CD34 and CD133, and MSCs, defined by the expression of CD105, CD73, and CD90, are attractive sources due to their autologous nature, ease of isolation and cryopreservation. The present review focuses on the use of bone marrow HSCs for liver regeneration, presenting evidence for an ongoing crosstalk between the hematopoietic and the hepatic system. This relationship commences during embryogenesis when the fetal liver emerges as the crossroads between the two systems converging the presence of different origins of cells (mesoderm and endoderm) in the same organ. Ample evidence indicates that the fetal liver supports the maturation and expansion of HSCs during development but also later on in life. Moreover, the fact that the adult liver remains one of the few sites for extramedullary hematopoiesis—albeit pathological—suggests that this relationship between the two systems is ongoing. Can, however, the hematopoietic system offer similar support to the liver? The majority of clinical studies using hematopoietic cell transplantation in patients with liver disease report favourable observations. The underlying mechanism—whether paracrine, fusion or transdifferentiation or a combination of the three—remains to be confirmed.

## 1. Introduction

The liver carries out vital physiological processes, including lipid metabolism, the detoxification of drugs, and the storage of glycogen to fuel the energy requirements of the body [1]. Despite advances in pharmaceutical drugs and surgery, orthotopic liver transplantation (OLT) remains the only curative option for advanced liver failure which constitutes a leading cause of death worldwide. Although it requires lifelong immunosuppression and may be associated with potential post-surgery complications and graft rejection/failure, OLT is the only intervention with proven long-lasting clinical benefit. However, the growing demand for donor organs requires alternative treatment options [2].

Hepatocyte transplantation has emerged as the first obvious choice for a cell-based strategy to restore liver function. Although considerable progress has been made with respect to techniques standardisation, major issues associated with cell engraftment and efficacy remain [3]. Additional cell transplantation strategies have been proposed with stem cells taking centre stage due to their self-renewal capacity and ability to differentiate into different cell types. Cells used in transplantation for liver therapy include endothelial progenitor cells (EPCs), fetal liver cells, hematopoietic stem cells (HSCs), induced pluripotent stem cells (iPSCs), and mesenchymal stromal cells (MSCs) [4,5]. Moreover, immune cell therapies for liver diseases are also available [6].

The rationale behind stem-cell-based therapies is to reverse the progress of liver failure by regenerating the liver tissue. The mechanisms underlying the regenerative capacity of stem cells could be multifactorial and mediated through the replenishment of damaged tissue, the provision of soluble factors or fusion with resident cells. The use of autologous rather than allogeneic stem cells for transplantation conveys immunological tolerance and eliminates the need for lifelong immunosuppression for the prevention of allograft rejection.

Mesenchymal stromal cells (MSC) have the potential to differentiate into adipocytes, chondrocytes and osteoblasts [7]. They can be isolated from various sources, including bone marrow, umbilical cord, placenta and amniotic fluid [8]. One of their main advantages is that they can be significantly expanded in culture under carefully controlled conditions to give rise to a large number of cells for transplantation. MSC-based transplantation in patients with chronic liver disease improves liver function, especially in the first months following administration [9] with minimal side effects [10]. The paracrine and immunomodulatory properties of MSCs exert a regenerative effect on many tissues, including the liver, by increasing angiogenesis and reducing oxidative stress and apoptosis [11]. Moreover, MSCs can differentiate into hepatocytes and BECs in vitro [12] and in vivo, restoring the damaged liver [13], while their extracellular vesicles (EVs) may also prove beneficial in liver therapy [14,15]. Numerous clinical trials have utilised MSCs for transplantation in patients with liver failure or cirrhosis demonstrating improvement in liver parameters and function [5].

iPSCs emerge from the reprogramming of somatic cells using a combination of defined factors, such as Oct4, Sox2, Klf4, and c-Myc [16]. iPSCs can differentiate into hepatocyte-like cells [17] and constitute an attractive source of cells for transplantation, as they lack ethical restrictions and can be produced in large quantities. Hepatocyte-like cells engraft in mice [18], and their administration protects against liver injury [19]. iPSCs can also generate Kupfer cells [20] and produce EVs with antifibrotic effects in mouse models of liver injury [21]. However, challenges associated with the generation of iPSC cells [22,23] still warrant their use in clinical trials.

The discovery of bone-marrow-derived EPCs that circulate in the peripheral blood traces back in the 1990s [24]. EPCs are recruited from the bone marrow to areas of neovascularisation and, through paracrine factors, exert cytoprotective effects and ameliorate fibrosis following transplantation in rats [25]. EPCs are increased in patients with liver cirrhosis [26], and administration in animal models of liver injury and cirrhosis increases hepatocyte proliferation and improves survival [27,28]. The transplantation of autologous EPCs in patients with liver cirrhosis (NCT01333228) was well tolerated and improved liver function, underscoring the therapeutic potential of these cells [29]. The procedure involves the short-term culture of bone marrow mononuclear cells (MNCs) on fibronectin in the presence of specific cytokines prior to transplantation.

Hepatocytes constitute the obvious cell choice for transplantation and a promising, minimally invasive alternative to OLT, as they overcome the need for complex surgery and lifelong immunosuppression. Adult hepatocytes are usually isolated from whole donor livers that have been rejected for OLT; one of the main advantages is the potential of treating multiple patients from a single donor [3]. The transplantation of hepatocytes in patients with liver failure seems to reduce mortality [30,31] and also serves as a bridge to OLT [32]. Experimental data indicate that the outcome of transplantation depends on the level of hepatocyte engraftment, and metabolic diseases offer a selective advantage to transplanted cells [33,34]. A recent approach involving the transplantation of human hepatocytes encapsulated in alginate microbeads seems to address the limitation of engraftment level; 8 children receiving intraperitoneal transplant with microencapsulated liver cells demonstrated clinical improvement, and 4 of them avoided OLT [35]. Overall, the limited availability of high-quality hepatocytes and damage following cryopreservation prohibits their widespread clinical utilisation. Fetal hepatocytes isolated from embryos have been employed as an alternative source of hepatocytes for transplantation with promising results [36,37].

The present review focuses on the use of HSCs as a cell source for liver therapy, recording the available clinical trials (case-control and randomised studies) using these cells in patients with different types of liver diseases. HSCs have been under investigation for over 60 years, and a lot of information is available regarding their identification, prospective isolation and biological properties, especially with regards to the reconstitution of the hematopoietic system. HSCs are the basis of bone marrow transplantation, a curative therapy for hematological malignancies, aplastic anemia and primary immunodeficiencies. One of the main advantages of using HSCs for transplantation is that recent pharmacological advances facilitate the mobilisation of autologous HSCs in the systemic circulation, overcoming the need for invasive procedures [38]. Moreover, minimal ex vivo manipulation is required and no in vitro culture, such as in the cases of MSCs or EPCs. At the same time, the inability to expand HSCs in vitro limits the number of cells that can be transplanted.

## 2. Hematopoietic System

### 2.1. Hematopoietic Stem Cells (HSCs)

Decades of research have generated important information on the biology, function and the supporting microenvironment of HSCs. These cells represent a rare (<0.01% of the total bone marrow) multipotent population that lies at the apex of the hematopoietic system and is gifted with a self-renewal capacity, which ensures that a stem cell population is maintained throughout life. A finely regulated balance between differentiation and self-renewal generates all the blood cell lineages in the adult hematopoietic system and frequently replenishes short-lived blood lineages, such as neutrophils and platelets, to sustain blood system homeostasis [39].

**Figure 1:**
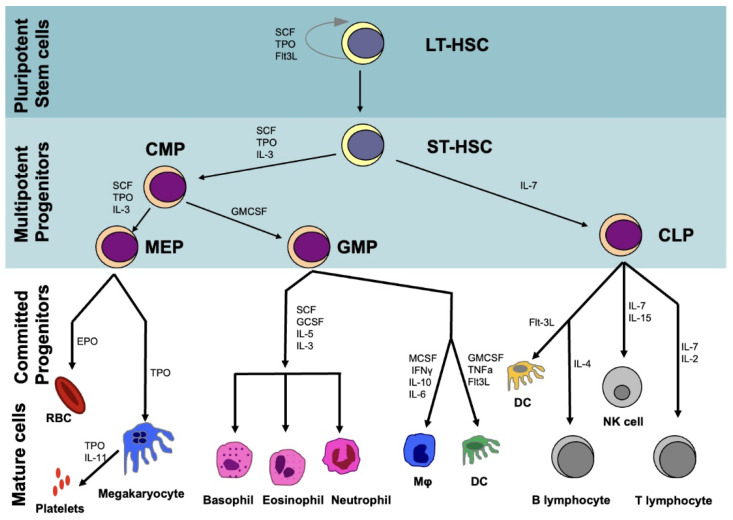
Outline of the hierarchy in the hematopoietic system. HSCs reside at the apex of the hematopoietic system and through a dynamically regulated process self-renew or differentiate to gradually give rise to multipotent progenitors (CMPs, CLPs). These in turn produce GMPs and MEPs and differentiate into committed progenitors and mature blood cells. LT-HSCs = long-term HSCs, ST-HSCs = short-term HSCs, CMPs = common myeloid progenitors, CLPs = common lymphoid progenitors, GMPs = granulocyte-macrophage progenitors, MEPs = megakaryocyte-erythrocyte progenitors, DC = Dendritic cells, Mφ = Macrophage, SCF = Stem cell factor, TPO = Thrombopoietin, Flt3L = Flt3 Ligand, IL = Interleukin, GMCSF = Granulocyte macrophage colony stimulating factor, MCSF = macrophage colony-stimulating factor.

HSCs are operationally defined by their ability to reconstitute the hematopoietic system upon myeloablation. Evidence for this capacity has emerged from transplantation studies in lethally irradiated mice. HSCs differentiate initially to produce long-term (LT-HSCs) and short-term HSCs (ST-HSCs) that differ in their self-renewal capacity and lineage commitment (Figure 1). ST-HSCs differentiate into hematopoietic progenitor cells, which subsequently give rise to common myeloid progenitors (CMPs) and common lymphoid progenitors (CLPs). CMPs produce granulocyte–macrophage progenitors (GMPs) and megakaryocyte–erythrocyte progenitors (MEPs). GMPs generate granulocytes, monocytes, and dendritic cells, while MEPs form erythrocytes and megakaryocytes. CLPs are responsible for producing T and B lymphocytes. Both progenitor cells (CMPs, CLPs, GMPs, MEPs) as well as terminally differentiated blood cells lack any self-renewal capacity [40].

The vast majority of HSCs are dormant, residing at the G0 phase, and have the unique ability to undergo both symmetric and asymmetric division, producing an identical HSC and a progenitor cell. The dynamics of the organisation of the hematopoietic system are carefully regulated through the complex interplay of intrinsic and extrinsic factors. Intrinsic factors responsible for maintaining HSC self-renewal include transcription factors [Runx1, GFI1, Scl, GATA2, EVI1] [41], epigenetic regulators [TET2, DNMT3A, EZH1] [42] and miRNAs [43]. Both human and mouse HSC seem to share a common miRNA signature (miR-125a, miR-125b, miR-155, miR-99a, miR-126, miR-196b, miR130a, miR-542, miR-181, miR-193, and miR-let7e), suggesting an evolutionary conservation of these molecules [44]. Extrinsic factors include the low levels of oxygen of the bone marrow microenvironment that HSCs are exposed to [45], although the hypoxic profile of HSCs also depends on cell-intrinsic mechanisms [43,46]. Key cytokines implicated in HSC self-renewal and maintenance include stem cell factor (SCF), thrombopoietin (TPO) and C-X-C motif chemokine ligand 12 (CXCL12) also known as stromal cell-derived factor 1 (SDF-1), a key chemokine responsible for also attracting adult HSCs to the bone marrow. Notch ligands may drive proliferation, while quiescence is maintained through osteopontin and TPO [47]. Canonical Wnt signalling, which is mediated via beta catenin, also regulates HSC self-renewal and differentiation capacity in a dose-dependent manner. HSC function and repopulation capacity require the mild activation of Wnt signalling, while higher levels promote T-cell differentiation [48].

**Figure 2:**
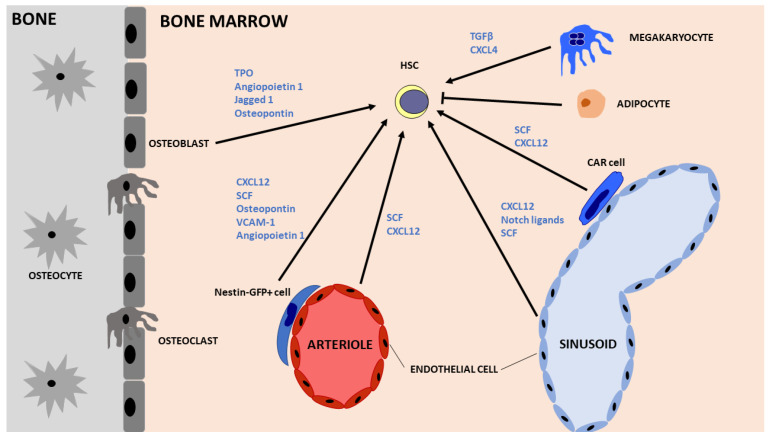
Schematic representation of the bone marrow niche showing the main components and key cytokines/factors implicated in HSC maintenance and self-renewal. HSCs have been found to localise close to arterioles and sinusoids (vascular niche) as well as close to the endosteum (endosteal niche). Nestin-GFP+ cells are perivascular cells highly enriched in MSCs while CXCL12-abundant reticular (CAR) stromal cells are adipo-osteogenic progenitors located in close proximity to sinusoids. HSC = Hematopoietic stem cell, TPO = Thrombopoietin, SCF = Stem cell factor, CXCL12 = C-X-C motif chemokine ligand 12, TGF-β = Transforming growth factor beta, CXCL4 = C-X-C motif chemokine ligand 4.

In the adult, the bone marrow serves as the site of hematopoiesis, with HSCs residing at specialised locations: the endosteal and vascular niches. The endosteal niche is located in close proximity to the trabecular bone and encompasses osteoblasts, endothelial cells, MSCs, megakaryocytes and adipocytes (Figure 2). The vascular niche is located adjacent to extravascular spaces of the bone marrow and is composed of endothelial cells, CXCL12-abundant reticular (CAR) cells and Nestin + GFP cells, which are enriched in MSCs [49]. Collectively, these sites and cells contribute to HSC maintenance, regulation and operational competence.

The prospective isolation and identification of HSCs is central for HSC transplantation and usually involves a combination of surface markers by flow cytometry analysis and sorting. Mouse HSCs are defined by the absence of blood lineage markers (Lin^−^), while they express c-Kit, Sca1 and lack CD34 expression. Human HSC markers include Lin^−^, CD34^+^, CD38^−^ and CD90^+^, while further isolation on the basis of CD49f expression enriches for HSCs [50]. Although a rare population of HSCs may lack CD34 expression [51], a recent study has identified endothelial protein C receptor (EPCR) to be expressed in HSCs with high repopulating and self-renewal ability. EPCR^+^ HSCs represent a pure HSC population with a stem cell frequency of 1 in 3 cells [52]. Separation on the basis of the surface CD33 marker has also been proposed for human HSCs [53].

### 2.2. Embryonic Hematopoiesis

During development, embryonic hematopoiesis occurs in three distinct waves involving the fetal liver as a transient hematopoietic site. Understanding the mechanisms by which the hematopoietic and hepatic systems crosstalk and potentially modulate each other during embryogenesis is central for improving their homing to the liver during transplantation in liver therapy. They may also provide important insight towards novel in vitro HSC expansion strategies.

The first hematopoietic cells emerge as blood islands in the yolk sac at around E7.5 in mice and 2–3 weeks post-conception in humans [54,55]. These primitive erythroid progenitor cells are accompanied by primitive megakaryocytes and macrophages. The former display a limited lifespan serving to transport oxygen to the developing embryo [56]. In contrast, macrophages are long-lived cells that can be encountered through adulthood in various tissues. A prime example is microglia, the resident macrophages of the brain that colonise the developing brain as early as E9 [57].

The second wave of de novo generation of hematopoietic progenitor cells with definitive erythroid and myeloid potential appears in the endothelium of the yolk sac shortly after the first wave at approximately E8.5 in the mouse. These cells maintain erythropoiesis until birth [58] but lack any definitive HSC activity [59].

The first bona-fide HSCs emerge in the aorta–gonads–mesonephros (AGM) region [60] at the floor of the dorsal aorta from hemogenic endothelium around E9.5–E11 in mice and 4 weeks post-conception in humans [61] and bud off to enter the vascular network [62]. These cells display HSC activity and can provide long-term multilineage hematopoietic reconstitution upon transplantation in animal models [63]. The three overlapping waves of embryonic hematopoiesis consisting of hematopoietic progenitors and definitive HSCs home and colonise the fetal liver at E11.5 in mouse and around 6 weeks post conception in humans. HSCs and other progenitors are also found in the fetal spleen after E15.5 and in the bone marrow at E17.5 [64,65].

## 3. Fetal Liver Crosstalk with HSCs

What attracts hematopoietic progenitors to the fetal liver? Are there any specific rules of attraction or environmental cues that drive fetal liver colonisation, or is it simply a matter of tissue architectural layout? Undoubtedly, the fetal liver resides at an anatomically privileged location within the embryo, constituting the first organ that HSCs encounter when travelling in the circulation. Additionally, at the time of colonisation, the hepatic sinusoids—the vascular structures of the fetal liver—are wide, potentially facilitating the access of hematopoietic progenitors to the fetal liver.

Nonetheless, there are certain signals that serve to either passively retain or actively attract HSCs in the fetal liver. Hematopoietic progenitors express vascular-endothelial cadherin (VE-Cadherin) alongside various integrins, selectins and CD44. The β1 integrin plays a key role in the ability of HSCs to colonise the liver [66]. The retention of hematopoietic progenitors and HSCs in the fetal liver is further mediated through association with the extracellular matrix (ECM) and in particular with fibronectin [67]. Additional cell adhesion molecules, such as vascular adhesion molecule 1 (VCAM-1), present on the surface of HSCs favour the interaction with fetal liver stromal cells and their in-tissue retention [65]. In situ imaging of HSC localisation in the fetal liver indicates close proximity to VE-Cadherin-expressing cells [68]. CXCL12 is instrumental to the retention of HSCs in the fetal liver, but, however, does not serve as a chemoattractant for initial fetal liver colonisation by HSCs [69].

During their transient residence in the fetal liver, HSCs undergo maturation and great expansion and obtain the capacity for long-term multilineage hematopoietic reconstitution upon transplantation [63]. Once in the fetal liver, HSCs expand 38-fold from E12 to E16 [70]. This indicates that the microenvironment of the fetal liver can provide the necessary support through growth factors and cytokines and potentially serves as a transient hematopoietic niche. Hepatoblasts secrete interleukin-7 and erythropoietin, controlling the proliferation and differentiation of lymphoid and erythroid progenitors, respectively. TPO produced by fetal liver hepatoblasts does not only promote megakaryocyte differentiation but also supports the survival of HSCs [71]. Embryos lacking the TPO receptor Mpl present with delayed HSC onset and reduced HSC self-renewal potential [72]. Additional molecules expressed by fetal liver cells, such as insulin-growth factor 2 [73], Flt3 ligand and KIT ligand, are important for sustaining the reconstitution potential of HSCs [65]. Furthermore, the ability of fetal liver cells to support HSC expansion is evident from co-culture studies of HSCs with hepatic progenitors positive for Delta-like 1 protein (DLK). This effect is mediated through physical contact and cannot be achieved using a conditioned medium nor DLK- fetal liver cells [74].

The fetal liver serves as a hematopoietic organ until the early postnatal period when the fetal spleen and the bone marrow take over with an HSC pool capable of sustaining hematopoiesis throughout their lifetime. This transition to the bone marrow goes in hand with a switch to a quiescent HSC phenotype [75].

Under pathological conditions in adulthood, such as bone marrow failure or myelofibrosis, HSCs leave the bone marrow and localise in distinct sites, where they continue to produce blood cells. The liver may serve as a site for such extramedullary hematopoiesis, keeping some principles of the fetal liver niche [65]. Additionally, liver sinusoidal endothelial cells support the in vitro B cell differentiation from HSCs [76]. This supports the concept of a continuous crosstalk between the hepatic and hematopoietic systems, even in the adult organism.

## 4. Inherent Liver Regeneration

The liver contains different cell types: parenchymal cells (<80% of liver mass), such as hepatocytes and non-parenchymal cells (20–40% of liver mass), which include liver sinusoidal endothelial cells (LSECs), biliary epithelial cells (BECs), Kupffer cells, and hepatic stellate cells, as well as various immune cells. Resection or acute injury of the liver induces a regenerative process that is mainly driven by hepatocytes, while in chronic liver diseases, hepatocyte progenitor cells are involved in regeneration [77]. Typically, liver regeneration comprises three phases: initiation, proliferation and termination. Innate immunity through complement activation is central for the initiation of liver regeneration [78]. The earliest biochemical signal in the regenerating liver is the urokinase plasminogen activator, which initiates a cascade of events involving metalloproteinases that break down the extracellular matrix and cause its remodelling [79]. Bile acid production is also a key event for the initiation and acceleration of liver regeneration [80]. During the initiation stage, hepatocytes enter the G1 phase of the cell cycle. This is triggered by the pro-inflammatory cytokines TNF-α and IL-6, which are produced by Kupffer cells. IL-6 activates the JAK/STAT, mitogen-activated protein kinase (MAPK) and PI3K signalling pathways in hepatocytes inducing regeneration. TNF-α activates the NF-κβ signalling and JNK pathway to trigger cyclin-dependent transcription in hepatocytes [81,82].

During the proliferation phase, hepatocytes transition into the mitotic phase of the cell cycle. This is initiated by hepatocyte growth factor (HGF), transforming growth factor alpha (TGF-α) and epidermal growth factor (EGF), which, in turn, initiate JAK/STAT, mitogen-activated protein kinase (MAPK) and PI3K signalling pathways to promote DNA synthesis and hepatocyte proliferation [83]. Additionally, fibroblast growth factor (FGF) [84], vascular endothelial growth factor (VEGF) [85], and insulin-like growth factor (IGF) [86] also support hepatocyte proliferation. The Wnt signalling pathway is another major contributor to hepatic regeneration, supporting hepatocyte proliferation through the expression of target genes, such as the cell-cycle regulator cyclin D1 [87]. Animals lacking beta catenin present with an inability to regenerate their liver [88]. Notch signalling also contributes to liver regeneration by supporting liver sinusoid endothelial cells to revascularize the liver parenchyma following liver insult [89]. Furthermore, angiogenesis takes place during this phase, with nonparenchymal cells undergoing proliferation in response to signals derived from proliferating hepatocytes [82].

**Figure 3:**
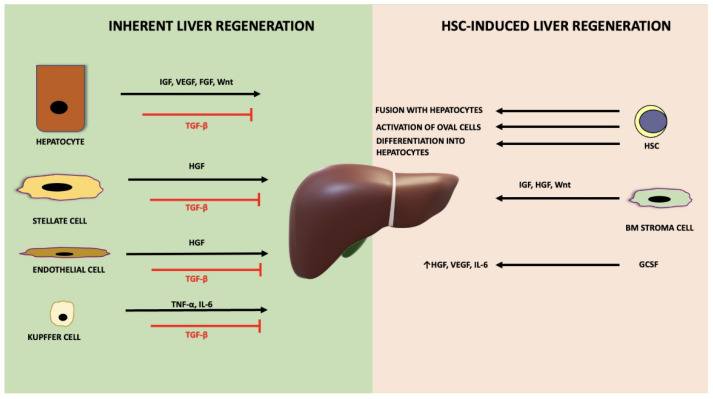
The mechanisms of inherent versus HSC-induced liver regeneration. Information from animal studies have indicated the contribution of various liver cell types (hepatocytes, stellate cells, endothelial cells, Kupffer cells) and factors (HGF, IGF, VEGF, FGF, TNF-α, IL-6, Wnt factors) in inherent liver regeneration. The hematopoietic system contributes to liver regeneration through factor secretion, activation of oval cells, differentiation into hepatocytes and fusion of HSCs with resident hepatocytes. GCSF also has an impact on liver regeneration by increasing HGF, VEGF and IL-6 levels. IGF = insulin growth factor, HGF = hepatocyte growth factor, VEGF = vascular endothelial growth factor, TNF-α = tumour necrosis factor alpha, IL-6 = interleukin 6 (IL-6), TGF-β = transforming growth factor beta, FGF = fibroblast growth factor, GCSF = granulocyte-colony stimulating factor.

When the required liver mass is achieved, the termination phase ensues, and hepatocytes stop proliferating. Various inhibitory molecules participate in this stage, such as IL-1 and IL-6 [82]. Transforming growth factor beta (TGF-β) also serves as a negative regulator of liver growth, and the disruption of TGF-β signalling in hepatocytes affects hepatocyte proliferation (Figure 3) [90]. HNF4-α known to regulate hepatocyte differentiation, promote the termination of liver regeneration [91], by reducing fibrosis [92]. The Hippo signalling pathway plays a central role in this final stage by regulating organ growth [93].

The process of liver resection is believed to trigger HSC mobilisation in the systemic circulation and homing to the liver itself around the sinusoidal space [94,95]. This seems to represent a spontaneous mechanism of HSC migration to sites of injury that are not restricted to the liver. Chemoattraction relies on the SDF-1 expressed by liver bile duct epithelium. Other stress-induced signals include MMP-9 and HGF [96]. Once in the damaged liver, HSCs attach to hepatic sinusoidal endothelial cells and BECs through integrins and CD44 [97]. Experimental evidence also pinpoints to an anti-fibrotic effect of HSCs on the injured liver [98].

Undoubtedly, animal models have provided valuable insight into the process, timing and signalling events that participate in liver regeneration. With human observational studies providing little information on the mechanisms of liver regeneration, there is a need for improved non-invasive methods to visualise tissue architecture and biochemical changes in regenerating the human liver [99].

## 5. HSC-Mediated Liver Regeneration

In the late 1990s to early 2000s, a number of studies indicated that bone marrow cells and HSCs are endowed with plasticity and can give rise to other cell types, including hepatic oval cells [100]. The study by Lagasse et al. indicated that HSCs had the ability to differentiate into hepatocytes upon infusion into animals with progressive liver failure, contradicting existing conceptions about the unilineage potential of HSCs. Researchers tracked expression of the LacZ transgene and identified distinct liver nodules of donor-derived cells alongside improvement in liver function parameters in animals transplanted with bone marrow HSCs [101]. Similarly, the liver of patients receiving sex-mismatched bone marrow transplant unveiled the presence of Y chromosome in 0.5–2% of hepatocytes [102,103].

The implied plasticity of HSCs was soon questioned; Wagers et al. (2002) used GFP as the tracking marker of HSC transplantation in lethally irradiated mice but failed to identify any contribution to non-hematopoietic tissues. This suggested that a strong selective pressure, such as metabolic deficiency, might be responsible for the previously observed HSC transdifferentiation or fusion events [104].

Later evidence based on the genetic analysis of individual hepatocytes indicated that cell fusion rather than transdifferentiation was the mechanism by which HSCs contributed to liver regeneration and/or repair [105,106]. Both studies employed the same mouse model of fumarylacetoacetate hydrolase deficiency and demonstrated the presence of donor bone marrow cell alleles, consistent with the formation of polyploid cells. Similarly, the transplantation of human GFP^+^CD34^+^ cells in a humanised mouse model of liver damage produced GFP+ hepatocytes. Genetic analysis of micro-dissected hepatocytes demonstrated the presence of both human and murine genetic material, indicating the ability of human HSCs to fuse with resident hepatocytes [107].

However, specific molecular or environmental cues may drive HSC differentiation into hepatocytes. Bone marrow cells were shown to generate hepatic oval cells following sex-mismatched transplantation in a mouse model of induced liver injury [100], while the in vitro transdifferentiation capacity of myeloid cells into hepatocytes was further documented when hepatocyte nuclear factor 4 alpha (HNF4α) was used as the molecular switch [108]. Additional experimental evidence corroborating the conversion of HSCs into functional hepatocytes both in vitro and in vivo without fusion is available for both mouse [109,110] and human HSCs [111] as well as for MNCs from umbilical cord blood [112].

More recently, Pedone et al. investigated the dynamics of the liver regeneration process using a combination of modelling and experimental approaches. Results showed that liver regeneration after partial liver hepatectomy in the absence of any metabolic pressure is dependent on the recruitment and formation of hybrid hepatocyte/bone marrow cells. CXCR4 plays a central role in this process with animals lacking CXCR4 in bone marrow cells displaying a compromised liver regeneration capacity. Interestingly, researchers observed fusion events in 15% of liver cells 3 days after resection, a percentage that increased to 50% up to 3 weeks following surgery. The study was designed to address the dynamics of bone marrow cell recruitment in relation to liver regeneration and not determine the regenerative capacity of bone marrow cell subpopulations [113].

The potential of HSCs to trigger or participate in the regeneration of other tissues after injury is also exemplified by the spontaneous migration of bone marrow stem cells to the heart after myocardial infarction. CD34+ cells can be detected in ischemic regions of the heart, and through cell fusion and transdifferentiation, they give rise to cardiomyocytes in vivo and improve myocardial regeneration and function [65,114]. These mechanisms are shared with those seen during HSC-based liver regeneration.

With respect to the capacity of HSCs to form hepatocytes in a sheep model, the cell dose, timing of transplantation and cell source (bone marrow, cord blood, mobilised peripheral blood) seem to play a central role [115]. Moreover, HSCs may stimulate oval cells in the liver or activate hepatic progenitor cells and promote their differentiation. This seems to peak after 3 months following HSC infusion, indicating an indirect effect of HSCs on the liver [116]. The fact that oval cells express several hematopoietic surface markers, such as CD34, Sca1 and CD45, suggests that they could represent an intermediate stage between the hematopoietic and hepatocyte lineages with common signalling mechanisms [117].

It is noteworthy that not all transplantation experiments were performed with defined cell types. BM-MNC contains not just one cell type, but a mixture of many types, including MSC, HSC, endothelial progenitor and stromal cells. MSCs may exert a clearer therapeutic impact through the release of trophic factors that support hepatocyte growth, prevent apoptosis and promote angiogenesis [5]. Bone marrow stromal cells and arterial endothelial cells in the bone marrow secrete HGF [118], which has also been shown to support HSC regeneration [119]. CD34^+^ HSCs express c-met, the receptor for HGF, and display improved in vitro clonogenic capacity in the presence of HGF [120]. Single-cell transcriptomic analysis of bone marrow subpopulations indicate that adipo- and osteo-CAR cells alongside arterial endothelial cells and some mesenchymal cell types express CXCL12 and SCF, while arteriolar fibroblasts express the highest levels of IGF [121]. Collectively, these findings suggest that key growth factors necessary for liver regeneration could be provided by bone marrow MNCs.

Given that most HSC liver therapy clinical studies employ granulocyte colony-stimulating factor (GCSF) for HSC mobilisation, one needs to consider the paracrine effects of its administration. GCSF antagonises the CXCR4 and SDF-1 interaction and mobilises HSC from BM to peripheral circulation. GCSF infusion in patients is accompanied by an increase in the serum levels of HGF and VEGF, which may aid liver regeneration [122]. GCSF-mobilised patients also present with higher levels of IL-6 [123] and an increased number of CD34+ cells in the liver [124]. In a mouse model of hind limb ischemia, the combination of GCSF and HGF portrays a synergistic effect in increasing angiogenesis and vasculogenesis [125]. Moreover, the regenerating liver of rats produces GCSF while oval cells express the GCSF receptor and respond to the growth factor [126]. The CXCR4 receptor antagonist plerixafor used as an alternative HSC mobilisation agent also seems to upregulate expression of VEGF and reduce fibrosis in a mouse model of liver damage [127]. Collectively, HSC mobilisation agents seem to exert both a direct hepatotrophic effect and to recruit HSCs to the damaged liver.

## 6. Clinical Trials of HSC Transplantation or Mobilisation in Patients with Liver Disease

We conducted a literature review using the Pubmed database and the NIH clinical trials database (https://www.clinicaltrials.gov/, last accessed on 15 June 2022) to identify clinical studies reporting HSC therapy for advanced liver disease. The search was performed using specific keywords: HSC, hematopoietic cell, liver therapy, liver disease, stem cell therapy and transplantation. Our search disclosed clinical trials, case reports, meta-analysis reports, and systematic reviews, as well as retrospective cohort studies. The literature review was limited to human studies, but no restrictions were applied regarding publication date. According to the NIH (https://www.clinicaltrials.gov/ database, accessed on 15 June 2022), there are 32 registered trials that involve the transplantation of unfractionated bone marrow MNCs, HSCs or investigate the impact of mobilisation agents alone. The majority have unknown or completed clinical status. The randomised controlled trial (RCT) with identifier NCT03109236 is currently active and investigates the transplantation of bone marrow CD133^+^ HSCs in patients with decompensated liver cirrhosis.

Table 1 presents the experimental protocol and published outcomes of 29 clinical studies dating from 2006 to 2019, involving the transplantation of various types of autologous adult hematopoietic cells. There was significant heterogeneity in the cell therapy protocol employed in the different studies, which involved bone marrow cell mobilisation alone with the use of GCSF, and/or isolation and reinfusion of bone marrow mononuclear cells (MNCs) or fractionated HSCs. The majority of the studies (72%) used GCSF to mobilise bone marrow cells in the systemic circulation and then isolate HSCs on the basis of the CD34 surface marker (35% of studies) and, in certain cases, through the expression of the surface CD133 marker (14% of studies). Unfractionated MNCs were used in 45% of the cases, while Zekri et al. (2015) co-infused HSCs and MSCs [128].

Although mainly employed as a mobilising agent for HSCs, G-CSF alone without HSC isolation and administration seems to improve liver parameters and survival in patients with acute-on-chronic liver failure with mild side effects [124,131,156].

Studies conducted up to 2008 were mainly case-control or proof-of-concept studies, while the need for RCTs became evident from 2010 onwards. As seen in Table 1, the number of cells infused is quite variable, but all studies monitor liver function by measuring the levels of albumin, total bilirubin, alpha fetoprotein, aspartate aminotransferase, alanine aminotransferase and prothrombin activity. Primary outcomes include survival and mortality as well as adverse effects associated with cell transplantation. Secondary outcomes are based on the model for end-stage liver disease (MELD) score and the Child Turcotte Pugh for Cirrhosis Mortality (CTP) score.

Most studies report improvements in at least one of the aforementioned liver parameters (Table 1) with the exception of five studies (17%) that showed partial [147,150] or no overall benefit of cell transplantation [144,153]. In an RCT of patients with decompensated alcoholic liver diseases, Spahr et al. assessed the impact of bone marrow MNCs transplantation following GCSF administration, compared to patients receiving steroids alone. After 3 months, no significant differences between the two groups were observed, while an improvement to the baseline parameters was evident in both the control and treated groups. Adverse effects were equally distributed in the two groups of patients [144]. The recent Newsome et al. RCT involved patients with compensated liver cirrhosis who were either treated with standard care, GCSF alone or GCSF followed by autologous administration of CD133^+^ HSCs [153]. No differences in MELD scores were observed among the groups, while adverse effects were more frequent in patients treated with GCSF or GCSF and CD133^+^ HSCs. Potential reasons for the absence of any benefit may lie in the number of cells transplanted or the percentage of HSCs and MSC populations found in the bone marrow of patients.

A recent Chinese follow-up study investigated the long-term outcome of autologous bone marrow HSC transplantation in patients with decompensated cirrhosis, who were treated from 2005 to 2012 [155]. The study included 151 subjects who showed a significantly higher 10-year survival rate, compared to controls who did not receive HSC transplantation.

Considering the small number of patients enrolled in each of these studies, meta-analyses are necessary to combine the data and generate a larger sample size with greater statistical power. In such a recent meta-analysis on the impact of stem cell therapy for chronic liver disease, there was an indication for an overall improved survival and liver function following cell transplantation, while no adverse side effects were documented [92]. A total of 24 RCTs were included in the meta-analysis involving autologous or allogeneic transplants of bone marrow-derived cells (MSCs or MNCs), umbilical cord MSCs and peripheral blood MNCs. Patients demonstrated improvement in total bilirubin and albumin levels, while there was no change in the hepatic enzyme levels. Stem cells derived from the bone marrow exhibited superior therapeutic effects to those from umbilical cord, while studies transplanting BM-MNCs recorded a significant effect on liver function later (24 weeks post-transplantation) than earlier (12 weeks) with those seen with MSCs.

Another meta-analysis evaluating the effect of bone marrow stem cell transplantation as a treatment for liver cirrhosis showed improvement in various liver function parameters (albumin, total bilirubin, aspartate aminotransferase, prothrombin time and activity) as well as CTP and MELD scores [157]. Again, studies infusing MSCs were included in this analysis, making it difficult to draw any clear conclusions on the impact of HSCs alone. The number of bone marrow stem cells infused was raised as a key parameter for achieving significant improvement in liver function [158].

Overall, the transplantation of purified HSCs or bone marrow cells in patients with liver disease appears to be safe and clinically beneficial to patients. Although a direct comparison of data from clinical trials using HSCs versus total bone marrow MNCs would be advantageous, it is also important to determine the regenerative mechanisms employed by each cell type. The concomitant administration of GCSF or plerixafor are advantageous to the clinical outcome because they seem to improve liver histology and accelerate regeneration [159]. The transplantation of unfractionated bone marrow MNCs following mobilisation has the advantage of including all cell populations, including MSCs, EPCs and other stromal cells, alongside paracrine factors that may be beneficial for liver regeneration.

## 7. Discussion

Liver diseases (acute and chronic) affect millions of people worldwide and may progress to cirrhosis, liver failure and hepatocellular carcinoma. The only curative option is OLT, but associated limitations and complications have turned scientists to search for alternative solutions. Stem-cell-based strategies offer promising alternatives for liver therapy, and various clinical trials have been conducted infusing HSCs and/or MSCs in patients with advanced stage liver disease. Even though liver cells are derived from the endoderm, and blood cells emerge from embryonic mesoderm, evidence regarding the transdifferentiation potential of HSCs in the early 2000s suggested their regenerative potential for liver therapy.

The clinical trials utilising bone marrow MNCs or purified HSCs demonstrate safety and relative efficacy but, depending on the extent of the liver damage, may not be sufficient to completely regenerate the organ. This is of great relevance in liver cirrhosis when fibrosis is prominent, and the unique architecture of the hepatic lobule is lost. GCSF-mediated mobilisation routinely used to obtain autologous HSCs from the bone marrow of patients may act synergistically as a growth factor towards hepatic regeneration. Nonetheless, there is a need to better understand the mechanisms underlying the regenerative potential of HSCs and of the other cell types present in the bone marrow cell transplants. Determining the role of each cell type and identifying the paracrine components that contribute to liver regeneration will improve existing clinical protocols for patients suffering from liver disease. Importantly, it may help overcome challenges associated with the survival of transplanted cells through pre-treatment or pre-conditioning [160].

The fact that liver patients present with heterogeneous and multiple comorbidities complicate clinical outcome and safe conclusions. Despite efforts to optimise protocols and clinical endpoints, there is a need for more standardised approaches and RCTs with large numbers of patients to draw meaningful conclusions. It would also be useful to extensively characterise the secretome of the transplanted stem cells to ensure that it includes key therapeutic factors and avoids molecules with a negative effect on liver regeneration.

The complexity of liver functions perplexes the use of assistive devices to maintain homeostasis as supportive therapy until liver transplantation (bridge to transplant) or liver regeneration (bridge to recovery). However, HSC cell transplantation may serve to bridge the time until organ transplantation takes place or can be given concomitantly with artificial liver support systems which remove albumin-bound toxins that accumulate in liver failure [161].

As the quest towards liver therapies continues, emerging evidence indicates that strategies including acellular mediators, such as EVs, could be explored. EVs can be derived from progenitor cells, such as HSCs, and can be utilised for delivering specific signals to the dysfunctional liver. EVs facilitate the packaging of a wide variety of molecules including lipids, nucleic acids and proteins and reflect the characteristics of the parental cell. They may also include signalling activators, which may prove beneficial for therapy. EVs and exosomes from several types of cells, including hepatocytes, cholangiocytes, hepatic stellate cells, and Kupffer cells as well as stem cells, have been thoroughly characterised for their support in liver regeneration [162,163]. Moreover, EVs can be loaded with chemicals and exploited as drug delivery systems. However, issues associated with the adequate purification and controlled quality of EVs obscure the wide adoption of the technology and the extraction of robust conclusions.

## 8. Conclusions

Cellular therapies constitute promising alternatives to OLT for various liver diseases. Herein, we discuss recent advances in the therapeutic application of bone marrow HSCs for liver regeneration. Evidence on the crosstalk between the hepatic and hematopoietic systems since embryogenesis supports the rationale of using HSCs as a therapeutic modality in patients with liver diseases. Promising results from clinical trials so far further support HSC-based clinical therapies. Nonetheless, better insight is needed into the mechanisms governing the regenerative potential of HSCs to help establish improved and safer protocols for their administration, alone or adjuvant to other cellular and acellular products in the context of liver diseases.

## Figures and Tables

**Table 1:** Clinical studies using hematopoietic cells for transplantation in patients with liver disease.

Author	Year	Country	Condition	Design	Patients (Treated/Control)	Cell Type	Cells Injection	Follow-Up Period	Outcome
**Terai S.** [129]	2006	Japan	Liver cirrhosis	Case-control	9/0	BM-MNCs (94% CD45^+^)	5.2 × 10^9^	24 weeks	Improved liver function and ALB levels. A trend towards ascites improvement
**Yannaki E. [130]**	2006	Greece	Alcohol-induced liver cirrhosis	Case-control	2/0	Autologous BM-HSCs (mobilised CD34^+^)	2 × 10^6^/kg	120 weeks	Improvement of baseline CTP and MELD scores
**Gaia S.** [131]	2006	Italy	Severe liver cirrhosis	Case-control	8/0	GCSF mobilisation	N/A	32 weeks	Improvement of baseline CTP and MELD scores in 50% of patients
**Gordon M. [132]**	2006	UK	Chronic liver failure	Case-control	5/0	Autologous BM-HSCs (mobilised CD34^+^)	10^6^–2 × 10^8^	60 days	Ν/A
**Mohamadnejad M.** [133]	2007	Iran	Decompensated cirrhosis	Case-control	4/0	BM-HSCs	2.5–8 × 10^6^	24 weeks	Improvement in ALB levels in 2 patients and MELD scores in 1 patient.
**Lyra A.C.** [134]	2007	Brazil	Chronic liver disease	Case-control	10/0	BM-MNCs	10^8^	16 weeks	Overall improvement in ALB, TBIL and INR
**Yan L. [135]**	2007	China	HBV-related decompensated liver cirrhosis	Case-control	2/0	Autologous BM-HSCs (GCSF-mobilised)	10^7^–10^8^/kg	18 months	Improvement of baseline CTP score
**Levicar N.** [136] LCER No. 2004/6746	2008	UK	Chronic liver disease	Case-control	5/0	Autologous BM-HSCs (GCSF-mobilised CD34^+^)	10^6^–2 × 10^8^	18 months	Improvement in ALB and AFP levels
**Khan A.A.** [137]	2008	India	Liver cirrhosis	Case-control	4/0	Autologous BM-HSCs (GCSF-mobilised CD34^+^)	0.1 × 10^8^	26 weeks	Improvement of baseline CTP and MELD scores
**Han Y.** [138]	2008	China	HBV-related decompensated liver cirrhosis	RCT	20/20	Autologous BM-MNCs (GCSF-mobilised)	10^7^–10^8^/kg	6 months	Improved ALB and CTP score in patients receiving cell transplant
**Pai M.** [139]	2008	UK	Severe alcoholic liver cirrhosis	Case-control	9/0	Autologous BM-HSCs (GCSF-mobilised CD34^+^)	2.3 × 10^8^	3 months	Improved TBIL, ALT, AST and CTP score. Some improvement in ascites formation
**Salama H.** [140]	2010	Egypt	End-stage liver disease	RCT	90/50	Autologous BM-HSCs (mobilised CD34^+^ and CD133^+^)	0.5 × 10^8^	24 weeks	Improved liver function and ALB levels
**Salama H.** [141]	2010	Egypt	End-stage liver disease	Case-control	48	Autologous BM-HSCs (GCSF-mobilised CD34^+^)	1 × 10^9^	48 weeks	Decrease in ascites; Improvement in ALB, TBIL, INR, ALT
**Kim J.K.** [116]	2010	China	Advanced liver cirrhosis	Case-control	10/0	Autologous BM-MNCs (80% CD45^+^)	0.5–1.5 × 10^8^	6 months	Improvement in CPT score and ascites formation
**Lyra A.C.** [142]	2010	Brazil	Chronic liver disease	RCT	15/15	Autologous BM cells	3.8 × 10^8^	12 months	The MELD score remained stable in treated patients while it increased in the control group. Improvement in ALB and TBIL in the treated group
**Saito S.** [143]	2011	Japan	Alcoholic liver cirrhosis	Case-control	5/5	Autologous BM-MNCs	8.0–7.3 × 10^9^	24 weeks	Higher ALB and PTA; improved CTP score
**Garg V.** [124] NCT01036932	2012	India	Acute-on-chronic liver failure	RCT	23/24	GCSF mobilisation	N/A	2 months	Improvement in survival, CTP and MELD scores
**Spahr L.** [144] ISRCTN83972743	2013	Switzerland	Decompensated alcoholic liver disease	RCT	28/30	Autologous BM-MNCs (GCSF-mobilised)	0.47 ± 0.15 × 10^8^/kg	3 months	**No improvement in liver function**
**Bai Y.Q.** [145]	2014	China	HBV-related liver cirrhosis	Case-control	32/15	Autologous BM-MNCs	Not reported	24 months	Improvement in ALB, PTA, fibrinogen, PLT, TBIL and reduction of adverse effects
**Liu L.** [146]	2014	China	Hepatitis B and decompensated liver cirrhosis	RCT	40/37	Autologous BM-MNCs (GCSF-mobilised)	3.2 +/−1.6 × 10^11^	4 weeks	Improvement in serum AST, ALT, ALB, and TBIL levels
**Andreone P.** [147] NCT01025622	2015	Italy	End-stage liver disease	Case-control	12/0	Autologous BM-HSCs (GCSF-mobilised CD133^+^)	5 × 10^4^/kg up to 1 × 10^6^/kg	12 months	**Temporary improvement in MELD score; worsening of CTP score**
**Zekri A.R.** [128] NCT01729221	2015	Egypt	HCV-associated liver cirrhosis	RCT	60/30	Autologous BM-HSCs (mobilised CD34^+^) followed by MSC infusion	0.5 × 10^8^	52 weeks	Improvement in baseline CTP in 40% patients. Improvement in ALB, TBIL and INR
**Sharma M.** [148]	2015	India	Non-viral decompensated cirrhosis	RCT	22/23	Autologous BM-HSCs (GCSF-mobilised CD34^+^)	N/A	3 months	Improvement in serum creatinin and MELD scores
**Deng Q.** [149]	2015	China	HBV-related decompensated cirrhosis	RCT	33/35	Autologous BM-HSCs (GCSF-mobilised CD34^+^)	2–4 × 10^7^	48 weeks	improvements in liver function (ALB, PTA) and portal vein hemodynamics
**TayebH.** [150]	2015	Egypt	HCV-associated liver cirrhosis	RCT	10/10	Autologous BM-MNCs (GCSF-mobilised)	25 × 10^6^–191 × 10^6^	3 months	Improvement in ALB levels 1 month post BMT; γ-GT improvement at 3 months; Improved CTP score at 3 months; **No statistical improvement in any other liver parameter at 3 months**
**Mohamadnejad M.** [151]	2016	Iran	Decompensated cirrhosis	RCT	8 (CD133) 10 (MNC) 9 (CONTROL)	Autologous BM-CD133^+^ versus BM-MNCs	2–13 × 10^8^ MNC/2–7 10^6^ CD133+	12 months	Improved MELD score in the CD133+ group at 3 mo
**Yu S.J.** [152] NCT01503749	2016	Korea	Decompensated cirrhosis	RCT	3 (GSCF + CELLS) 3 (GCSF) 3 (CONTROL)	Autologous BM-MNCs (GCSF-mobilised)	1.67 × 10^9^–2 × 10^10^	6 months	**Small improvement in CTP scores at 24 weeks**
**Newsome** [153] ISRCTN 91288089	2018	UK	Compensated liver cirrhosis	RCT	28/26 GCSF/27 control	Autologous BM-HSCs (GCSF-mobilised CD133^+^)	0.2 × 10⁶	90 days	**No improvement in liver dysfunction or fibrosis while adverse events may occur compared with standard care**
**Esmaeilzadeh A.** [154] IRCT2014091919217N1	2019	Iran	Decompensated liver cirrhosis	RCT	10/10	Autologous BM-MNCs	8.06 ± 2.5×10^6^ cells/kg	6 months	Improvement in MELD socre, INR, TBIL, ALB levels after cell transplantation (6 months)
**Cui L.** [155]	2022	China	Decompensated liver cirrhosis	10 yr follow up study	287/151	PBSC			Survival was higher in treated group alongside ALB levels, CTP and MELD scores

N/A not available; ALB-albumin; TBIL-total bilirubin; AFP-alpha fetoprotein; INR-international normalized ration with respect to prothrombin time; PTA- prothrombin activity; ALT -alanine aminotransferase; AST-aspartate aminotransferase; RCT-randomised controlled trials; MELD score https://www.mdcalc.com/meld-score-model-end-stage-liver-disease-12-older (accessed on 10 June 2022); CTP score https://www.mdcalc.com/child-pugh-score-cirrhosis-mortality (accessed on 10 June 2022).
